# Recent advances in AI-driven deep eutectic solvent design for biocatalysis: Co-design of extremozyme and reaction medium

**DOI:** 10.1016/j.bidere.2026.100109

**Published:** 2026-09-10

**Authors:** Yuxuan Hou, Jinyu Meng, Binghui Chen, Jifeng Yuan, Shizhen Wang

**Affiliations:** a Department of Chemical and Biochemical Engineering, College of Chemistry and Chemical Engineering, Xiamen University, Xiamen, 361005, China; b School of Computing and Data Science, Xiamen University Malaysia, Sepang, Selangor, 43900, Malaysia; c Fujian Ocean Innovation Center, Xiamen, Fujian, 361102, China; d State Key Laboratory of Cellular Stress Biology, School of Life Sciences, Faculty of Medicine and Life Sciences, Xiamen University, Fujian, China; e School of Advanced Interdisciplinary Biomedical Sciences, Faculty of Medicine and Life Sciences, Xiamen University, Fujian, 361102, China; f School of Energy and Chemical Engineering, Xiamen University Malaysia, Sepang, Selangor, 43900, Malaysia

**Keywords:** Deep eutectic solvents, Biocatalysis, Artificial intelligence, Extremozymes, Solvent engineering

## Abstract

Deep eutectic solvents (DES) have emerged as sustainable, highly tunable alternatives to conventional organic media for non-aqueous biocatalysis. DES dynamically interact with enzymes to shift reaction equilibria, enhance thermostability and tailor stereoselectivity. However, the vast combinatorial space and complex physicochemical properties of DES make traditional trial-and-error solvent engineering highly inefficient. This review systematically examines recent advancements in DES-driven biocatalysis and highlights the role of artificial intelligence (AI) for rational DES design. Specifically, it synthesizes evidence that DES composition and water content jointly shape enzyme-specific microenvironments and thereby affect catalytic performance. Extremozymes are also evaluated as candidate catalysts for DES-containing media and as sources of potentially transferable adaptation features for enzyme engineering. It then discusses how AI can support DES formation screening, prediction of physicochemical properties using quantitative structure-property relationship (QSPR) models, and candidate formulation generation at the solvent level. At the system level, the focus shifts to predicting how a specific enzyme responds to a defined formulation in terms of activity and stability. Finally, we outline a mechanistically informed, multi-objective framework that integrates DES composition, enzyme sequence and structure, and operating conditions with physicochemical calculations, AI-based prediction, and experimental validation, providing a practical route toward genuine DES-enzyme co-design.

## Graphical abstract

**Figure:**
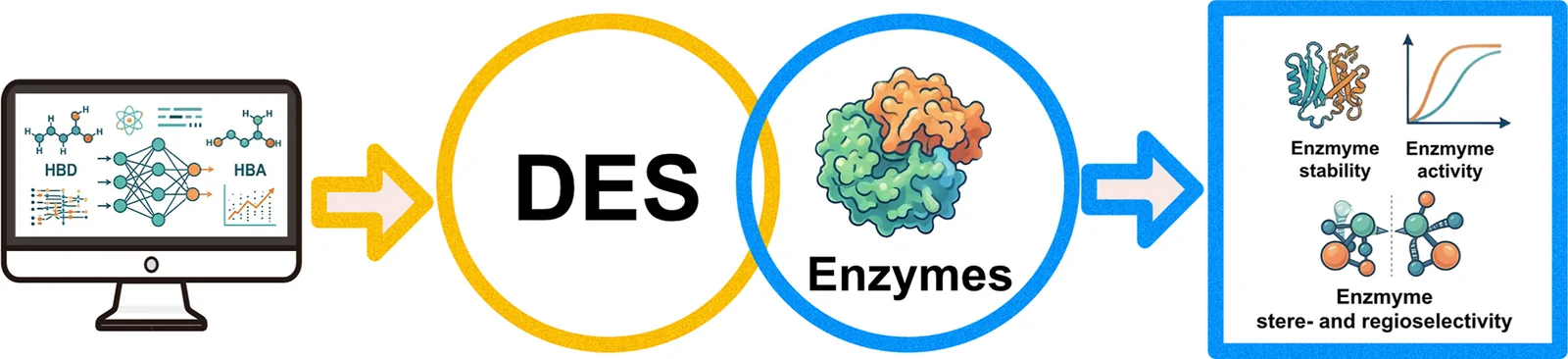

## 1 Introduction

Biocatalysis represents one of the most profound advancements in modern science [1]. Enzymes outstanding substrate specificity and chemo-, regio-, and stereoselectivity preclude the need for activation or protection steps, facilitating highly efficient cascade reactions with minimal waste [2].

Driven by the natural evolution of enzymes, conventional biocatalysis is primarily conducted in aqueous media [3]. Historically, enzymes were thought to require a bulk aqueous environment to preserve their active three-dimensional structures [4]. However, this aqueous requirement can be problematic for many industrially relevant substrates [5]. Coupled with the production of massive wastewater volumes [6], these drawbacks make traditional media highly unfavorable for such biocatalytic processes.

Over the past two decades, the focus of non-aqueous medium has gradually shifted toward sustainable, non-volatile solvent alternatives, such as ionic liquids (IL) and DES [7,8]. This transition is primarily driven by the severe drawbacks of traditional volatile organic compounds (VOC), which are highly volatile, flammable, inherently toxic to operators and pose massive environmental risks [9].

Among these alternatives, DES have recently gained prominence as innovative analogues to IL [10]. DES are typically formed through the physical complexation of a hydrogen bond acceptor (HBA) and a hydrogen bond donor (HBD). When mixed at a specific molar ratio, these solid or highly viscous precursors rapidly interact to form a dense, three-dimensional intermolecular hydrogen-bonding network [11,12], which macroscopically results in a melting point significantly lower than that of the individual precursors.

Although DES and IL share desirable macroscopic properties like negligible vapor pressure, high thermal stability and tunable polarity, DES are considered more environmentally sustainable due to their preparation from inexpensive and biodegradable components [13]. This inherent cost-effectiveness significantly enhances their potential for industrial-scale application.

Choi et al. further introduced the concept of natural deep eutectic solvents (NADES), which are composed entirely of natural components and exist ubiquitously in living organisms [14]. Specifically, many plants and extremely drought-resistant organisms typically accumulate high intracellular levels of specific primary metabolites such as simple sugars (glucose, sucrose), organic acids (malic acid, citric acid), amino acids, and choline, etc. These compounds can form NADES, which function as a distinct “third liquid phase” within living cells alongside water and lipids [14,15]. These properties have promoted the rapidly expanding application of DES in biocatalysis. Beyond solubilizing hydrophobic substrates, DES also enhance enzyme activity and stability and increase reaction yields [16,17].

However, despite these profound advantages, the broader implementation of DES-based biocatalytic systems remains constrained by several fundamental physicochemical and methodological limitations [18]. The traditional trial-and-error approach to DES screening and process engineering is no longer viable given the scale of the chemical design space [19]. AI-assisted strategies provide a rational route to prioritize candidate DES and link DES with activity, stability and selectivity, thereby reducing experimental workload and accelerating DES-enzyme system design [17,18,20]. Moreover, given their tolerance to extreme environments, extremozymes hold promise as important models for investigating DES-enzyme systems [21,22].

This review aims to summarize recent advances and limitations in the application of DES in biocatalysis and discusses the potential of AI-assisted strategies to guide the rational, data-driven co-design of DES media and enzymes.

## 2 Application of DES in biocatalysis

Currently, hydrophilic DES account for most cases of DES-enzyme synergistic systems. These systems are highly concentrated on choline salt- or betaine-polyol formulations. Commonly used polyols include glycerol, ethylene glycol, and sorbitol. One possible reason is that these formulations are readily miscible with water or buffer. This miscibility allows the system pH and viscosity to be adjusted in a targeted manner by varying the buffer conditions and aqueous fraction [18,23]. During DES-enzyme interactions, enzyme performance is jointly governed by the molecular properties of the individual DES components and the bulk physicochemical properties of the resulting medium. Among these factors, water activity and enzyme hydration are the most fundamental enzyme-related variables because they determine whether a functional protein hydration shell can be maintained [24,25]. The properties of the HBA and HBD may influence their chemical compatibility with the enzyme by affecting the ionic and hydrogen-bonding environment near the protein surface and active site [23,26]. At the transport level, viscosity and diffusivity regulate the mass transfer of substrates, products, and cofactors, thereby affecting the observed catalytic rate [18,27]. The pH and polarity of the medium are also important for particular enzymes and reactions because they affect active-site protonation, molecular charge states, substrate partitioning, and enzyme stability [28,29].

For a suitable DES-enzyme system, the central challenge is not merely the vast chemical space of possible DES formulations. It is whether a formulation creates an appropriate microenvironment for the enzyme. The most important feature of this microenvironment is the hydration state actually experienced by the enzyme. Most systems discussed in Section 2 are DES-water or DES-buffer mixtures. Many enzymes show reduced activity or even become inactive in neat DES or at high DES fractions. Adding water changes enzyme hydration as well as the DES hydrogen-bond network, viscosity, diffusivity, polarity, and the distribution of individual components around the protein surface [[23], [24], [25]]. Because a change in formulation simultaneously affects several coupled properties and competing performance endpoints, identifying the optimal enzyme microenvironment is a multi-objective optimization problem that is increasingly difficult to address through conventional experimental screening alone. Researchers have therefore begun to use data-driven and AI methods to convert empirical DES screening into enzyme-specific formulation ranking and performance prediction [17,18,30]. At present, these applications remain primarily forward screening within predefined candidate spaces rather than true inverse generative design.

The roles of DES in biocatalysis can be broadly categorized into three aspects: improving substrate solubility and enzyme activity, modulating enzyme stability, and tailoring stereo- and regioselectivity (Fig. 1) [31,32]. Accordingly, this review categorizes recent advances in DES-driven medium engineering and further introduces marine and extreme enzymes as environmentally tolerant enzyme models for DES-enzyme systems and discusses their implications for future solvent-enzyme co-design.

**Fig. 1:**
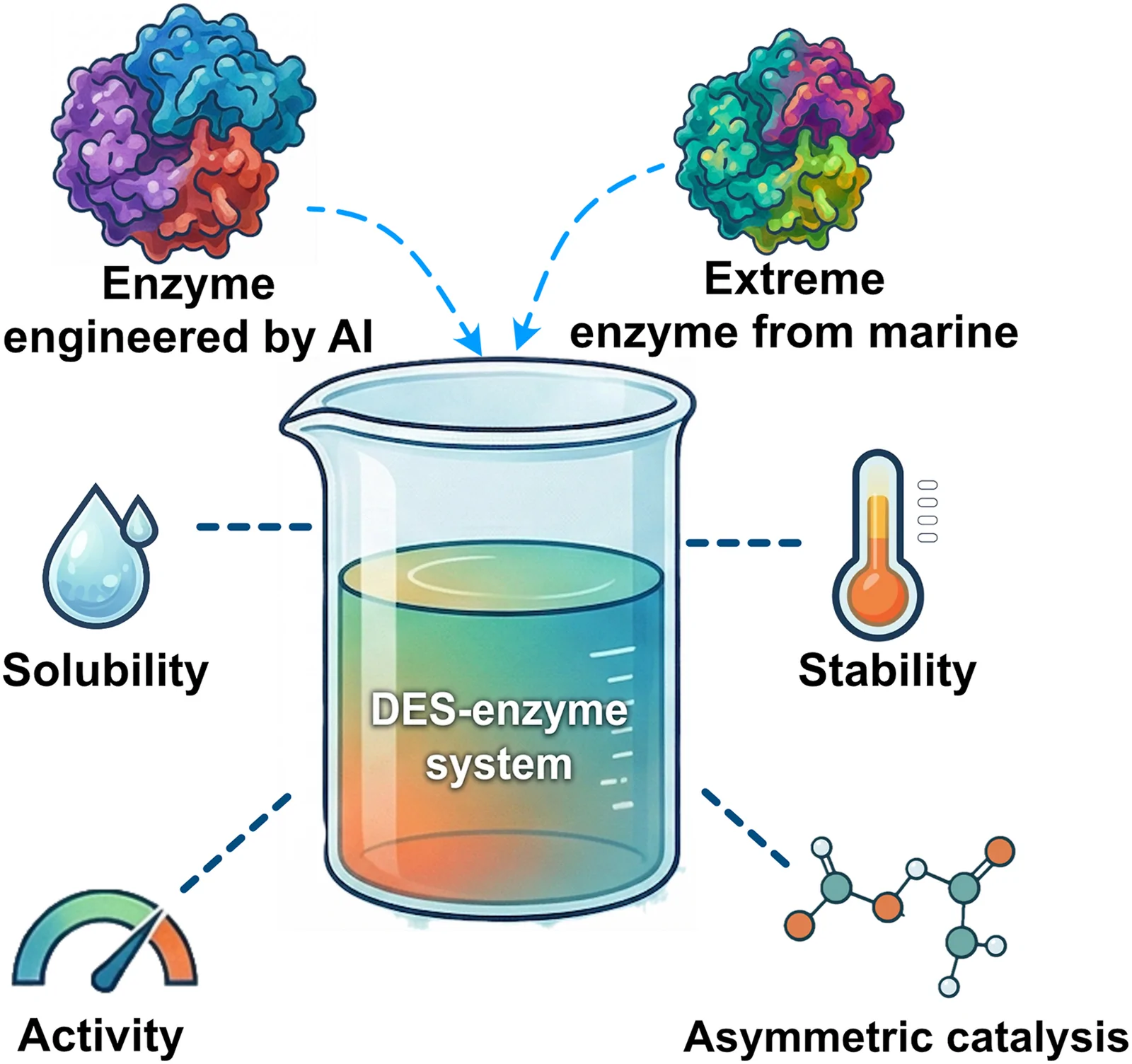
DES-enzyme co-design systems.

### 2.1 Modulating biocatalytic reaction performance

DES-containing media can enable biocatalytic reactions at high substrate loadings. Schweiger et al. demonstrated that a ChCl:glycerol DES (1:2) containing 50% water supported the decarboxylation of caffeic acid by *Bacillus subtilis* phenolic acid decarboxylase (BsPAD) at a substrate loading of 300 mM while maintaining more than 99% endpoint conversion [33]. Cao et al. found that dihydromyricetin (DMY) was 1.73-fold more soluble in the screened DES than in DMSO. Adjusting the DES:DMSO volume ratio from 3:1 to 1:3 increased DMY conversion from 18.8% to 91.3%, while the optimized medium gave an initial reaction rate of 11.2 mM h^−1^ [34].

Beyond reactant availability, the formulation must also preserve an enzyme-compatible microenvironment so that increased substrate availability can be translated into a high reaction rate, conversion, and ultimately product yield. Bittner et al. further demonstrated the importance of adjusting water content to maintain a suitable enzyme microenvironment. ChCl:Gly (1:9) containing 20% water provided sufficient substrate solubilization while maintaining the activity and stability of horse liver alcohol dehydrogenase. In this medium, the reduction of cinnamaldehyde to cinnamyl alcohol reached a productivity of 15.3 g L^−1^ d^−1^ [23]. Allan et al. coupled a [NiFeSe] hydrogenase from *Desulfomicrobium baculatum* with TiO_2_ for photocatalytic H_2_ evolution in aqueous glyceline (ChCl:glycerol 1: 2). Increasing the glyceline fraction reduced O_2_ diffusivity and limited O_2_ transport to the catalyst, thereby improving the operational oxygen tolerance of the hydrogenase, whereas increasing the aqueous fraction favored catalytic activity. A medium containing 60% glyceline provided the best balance between activity and oxygen tolerance, sustaining H_2_ evolution under air for 72 h with an overall turnover frequency of 60 s^−1^ [27].

DES can also enhance catalytic performance by regulating enzyme conformation. DES facilitated horseradish peroxidase (HRP) activity by promoting a higher α-helix content and a relaxed tertiary structure [26]. Furthermore, DES components can directly participate in reactions, forming a reactive deep eutectic solvent (RDES) [35]. Phospholipase D (PLD) catalyzes the transphosphatidylation (alcoholysis) of phosphatidylcholine (PC) in the presence of an alcohol (X-OH), which acts as a competing nucleophile to inhibit its hydrolysis into phosphatidic acid (PA) [36]. DES containing glycerol or ethylene glycol as HBD can create a hydroxyl-rich microenvironment, which suppress hydrolysis and improving the yield of phosphatidylserine in PLD-catalyzed transphosphatidylation [37].

The cases discussed above show that DES can modulate biocatalytic performance through several formulation-dependent routes, including increasing substrate availability, altering the enzyme microenvironment, and changing competing reaction pathways. AI-assisted screening should therefore be regarded as a hierarchical process rather than a single solubility-prediction task. Current AI applications mainly address the upstream feasibility of DES formation. Abbas et al. used molecular dynamics (MD)-derived hydrogen-bond numbers and lifetimes to train machine-learning classifiers that distinguished DES from non-DES systems, with the best extra trees forest model achieving an ROC-AUC of 0.88 in experimental validation [38]. Ayres et al. developed a Transformer-based binary classifier to predict whether candidate components and molar ratios would form stable NADES. The model identified 337 candidates predicted to form stable NADES, and 8 of the 10 highest-ranked ibuprofen-containing formulations formed stable mixtures experimentally [39]. However, these models primarily address the initial screening of DES formation and do not jointly predict substrate loading and catalytic performance or explicitly incorporate mechanistic variables such as enzyme conformation and hydration, mass transfer, or competing reaction pathways. Candidate formulations identified by AI-based virtual screening therefore still require experimental validation against performance endpoints relevant to the target biocatalytic function.

Table 1 highlights recent experimental studies in which DES-containing media enhanced biocatalytic performance by increasing enzyme activity, enabling higher substrate loadings, or improving substrate conversion.

**Table 1:** Recent studies on DES-enhanced enzyme performance.

Enzyme	DES composition	Aqueous phase	DES-induced effect	Reference
Subtilisin on chitosan	ChCl:Gly (1:2)	3% water	Reaction activity was nearly 6 times that in *t-*butanol containing 2% water	[40]
Enzyme system containing β-glucosidase and β-galactosidase	Bet:EG (1:2)	10% DES in 20 mM citric acid buffer (pH 6.0)	Increased deglycosylated ginsenoside conversion by 1.1-fold	[29]
Novozym 435, a commercial immobilized *Candida antarctica* lipase B	Choline acetate:Gly (1:1.5)	1.0% water and 20% methanol	Achieved 97% conversion after 3 h	[41]
Phenolic acid decarboxylase from *Bacillus subtilis*	ChCl:Gly (1:2)	50% water	Achieved 99% conversion of caffeic acid at a substrate loading of 300 mM	[33]

Note: Bet: betaine; ChCl: choline chloride; Gly: glycerol; EG: ethylene glycol.

### 2.2 Modulating enzyme thermostability

Most industrial enzymes are sourced from fungi or mesophilic bacteria, which limits their tolerance to the high-temperature of industrial processes [42,43]. Accumulating evidence indicates that DES can enhance enzyme thermostability while preserving catalytic activity.

The choice of HBA and HBD components can influence enzyme thermostability. For example, Kim et al. found that the thermostability of *Candida rugosa* lipase correlated with the HBD ability of the medium. In 0.1 M phosphate buffer (pH 7.0) containing 10% ChCl:Gly (1:2), the enzyme half-life at 40 °C increased from 1.0 to 9.2 h [28]. In contrast, Romano et al. showed that, for *Humicola insolens* cutinase, HBA identity made the largest contribution to the observed thermoprotection. A buffered medium containing 20% choline dihydrogen citrate:xylitol (1:1) increased the enzyme half-life at 90 °C from 22 to 62 min [44].

In addition, the aqueous fraction can affect the interactions between DES components and proteins and alter enzyme hydration, thereby further modulating enzyme thermostability. Chan et al. compared four DES formulations for *Myceliophthora thermophila* laccase. Among them, 5% lactic acid: betaine (2:1) increased the enzyme’s melting temperature (T_m_) from 86.7 to 88.5 °C, while 8% of the same DES increased catalytic activity to 300% of the control. Spectroscopic and crystallographic analyses further revealed DES-induced changes in enzyme conformation, hydration state, and the local environment of the active site [45]. Guo et al. examined ChCl-based DES containing 0-90% water. After incubation at 80 °C for 2 h, the residual activities of cellulase and pectinase first increased and then decreased as the water content increased [46]. MD simulations of selected formulations showed that the addition of a moderate amount of water increased the number of protein-solvent hydrogen bonds, reduced residue fluctuations, and produced a more compact enzyme structure. Overall, these studies indicate that enzyme thermostability in DES-enzyme systems depends on DES composition and aqueous fraction, which together modulate enzyme hydration and protein-solvent interactions.

Existing AI studies have modeled the time-dependent stability of enzymes in DES media at fixed moderate temperatures. Logarušić et al. used density, viscosity, pH, and σ-profile descriptors as candidate inputs for piecewise linear regression (PLR) models predicting FDH and NADH half-lives, with R^2^ values of 0.738 and 0.856, respectively [18]. Radović et al. calculated ten COSMO-RS mixture σ-profile descriptors incorporating the contributions of the HBA, HBD, and water. Enzyme-specific descriptor subsets were selected by correlation analysis and used in multilayer perceptron (MLP) neural networks to predict the inactivation rate constants of three dehydrogenases, with R^2^ values ranging from 0.893 to 0.990 [17].

Although both studies used time-dependent stability endpoints, these endpoints were measured at fixed temperatures, and temperature was not included as a model input. The resulting models therefore predict the effects of DES formulation on enzyme stability under the tested conditions rather than the temperature dependence of enzyme stability. Nevertheless, their use of mixture descriptors and kinetic stability endpoints provides a useful starting point for thermostability modeling. Extending this framework would require activity-decay measurements across multiple temperatures and the explicit inclusion of temperature during model development.

Table 2 summarizes recent representative studies utilizing DES to modulate enzyme thermostability, as evaluated by melting temperature, thermal-inactivation half-life, or residual activity after defined heat exposure. These studies show that the extent of thermoprotection depends on both the DES formulation and the enzyme examined. Therefore, DES composition, aqueous fraction, and buffer conditions should be jointly optimized to meet the microenvironmental requirements of the target enzyme.

**Table 2:** Recent applications of DES in modulating enzyme thermostability.

Enzyme	DES composition	Aqueous phase	DES-induced effect	Reference
*Myceliophthora thermophila* laccase	Lactic acid:Bet (2:1)	5% DES in 50 mM sodium acetate buffer (pH 5.0)	Increased the melting temperature from 86.7 to 88.5 °C	[45]
*Candida rugosa* lipase	ChCl:Gly (1:2)	10% DES in 0.1 M phosphate buffer (pH 7.0)	Extended the half-life at 40 °C from 1.0 to 9.2 h	[28]
*Humicola insolens* cutinase	ChDHC:Xyl (1:1)	20% DES in 0.1 M sodium phosphate buffer (pH 7.8)	Extended the half-life at 90 °C from 22 to 62 min	[44]
Cellulase and pectinase	ChCl:EG (1:2)	10% water	Retained approximately 113% cellulase activity and 190% pectinase activity after 2 h at 80 °C	[46]
POXA1b laccase	Bet:sorbitol (2:1)	25% DES in 50 mM phosphate buffer (pH 7.0)	Extended the half-life at 70 °C from 23 to 89 min	[47]

Note: Bet: betaine; ChCl: choline chloride; Gly: glycerol; ChDHC: choline dihydrogen citrate; EG: ethylene glycol; Xyl: xylitol.

### 2.3 DES induced tailoring of stereo- and regioselectivity

The majority of marketed drugs exist as single enantiomers, while the other may be inactive or even undesired. Enzymatic asymmetric catalysis has emerged as one of the most promising approaches for synthesizing chiral drugs and can now compete with traditional chemical methods [48,49]. Advances in biotechnology further allow enzymes to be produced and engineered on demand [50]. Therefore, modulate enzymatic selectivity by DES represents a highly promising strategy for enzymatic asymmetric catalysis.

Cicco et al. reported that ChCl:glycerol (1:2) and ChCl:sorbitol (1:1) DES-buffer mixtures enabled purified ketoreductases to achieve >99% conversion and >99% enantiomeric excess (ee) in asymmetric ketone bioreduction [51]. Vitale et al. demonstrated solvent-controlled stereoinversion in the baker's-yeast-mediated reduction of arylpropanones [52]. Specifically, switching from water to aqueous DES mixtures inverted the stereochemical outcome from the *S*- to the *R*-alcohol with up to 98:2 for both enantiomers. These representative DES-mediated effects on enzymatic selectivity are summarized in Fig. 2.

**Fig. 2:**
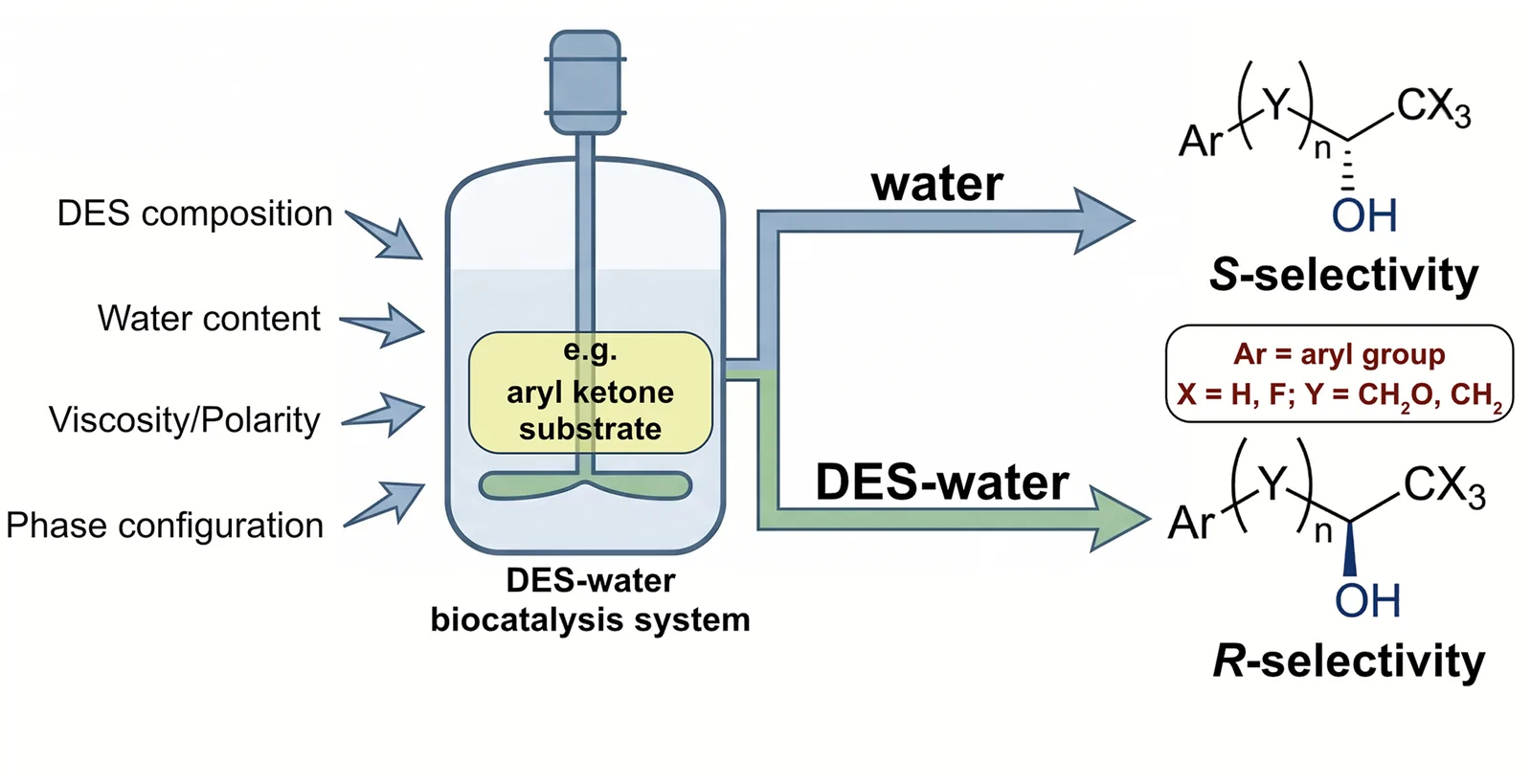
The overview of DES-mediated modulation of enzymatic stereoselectivity, using aryl ketone reduction as a representative example.

In addition to stereoselectivity, DES can also alter regioselectivity. Lindberg et al. showed that ChCl:glycerol or ChCl:ethylene glycol shifted the regioselectivity of potato epoxide hydrolase StEH1 in trans-2-methylstyrene oxide hydrolysis, increasing the regioselectivity ratio from 1.35 in buffer to 2.33 [53]. Table 3 summarizes representative applications of DES-mediated selectivity modulation in reduction, oxidation, hydrolysis, and bioamination reactions.

**Table 3:** Representative applications of DES in tailoring enzymatic stereo- and regioselectivity.

Reaction	Biocatalyst	DES composition	Aqueous phase	DES-induced effect	Reference
Stereoselective reduction	Purified ketoreductases (KRED)	ChCl:Gly (1:2) or ChCl:sorbitol (1:1)	20-50% 125 mM phosphate buffer (pH 7.0)	Achieved >99% conversion and >99% ee for secondary alcohols	[51]
Stereoselective reduction	Baker’s yeast whole cells	ChCl:Gly (2:1)	10% water	Reversed stereopreference from *S*- to *R*-alcohol; up to 98:2 er in either direction	[52]
Regioselective hydrolysis	Potato epoxide hydrolase StEH1	ChCl:EG (1:2)	40% DES in phosphate buffer	Increased regioselectivity ratio from 1.35 in buffer to 2.33	[53]
Asymmetric hydrolysis	Soybean epoxide hydrolase immobilized on UiO-66-NH_2_ MOF	ChCl:urea (1:2)	15% DES in PBS	Achieved 41.2% yield and 84.7% ee for (*R*)-1,2-octanediol	[54]
Asymmetric oxidation	Immobilized *Acetobacter* sp. CCTCC M209061 cells	ChCl:Gly (1:2)	72% 100 mM TEA-HCl buffer (pH 6.5); 20% [C_4_MIM][PF_6_] as the second phase	Achieved 50.2% conversion and 99.9% ee of residual (*S*)-MOPE at the 500 mL scale.	[55]
Stereoselective reduction	*Acetobacterpasteurianus* GIM1.158 cells	ChCl:EG (1:2)	48% 50 mM TEA-HCl buffer (pH 5.0); 20% [C_4_MIM][PF_6_] as the second phase	Achieving >99.9% ee for (*R*)-2-octanol	[56]
Asymmetric bioamination	Amine transaminase from *Exophiala xenobiotica*	ChCl:Gly (1:2)	Approximately 90% aqueous phase during bioamination	Achieved (*R*)-biaryl amines with >99% ee	[57]

Note: ee: enantiomeric excess; er: enantiomeric ratio; ChCl: choline chloride; Gly: glycerol; EG: ethylene glycol; IL: ionic liquid; KRED: ketoreductase; MOPE: 1-(4-methoxyphenyl)ethanol; MOF: metal–organic framework; PBS: phosphate-buffered saline; TEA–HCl: triethanolamine hydrochloride; [C_4_MIM][PF_6_]: 1-Butyl-3-methylimidazolium hexafluorophosphate.

Although these proof-of-concept studies demonstrate considerable potential, the implementation of DES-mediated asymmetric biocatalysis in chemical and pharmaceutical manufacturing remains limited [31]. To our knowledge, no mature QSPR models have yet been reported for predicting ee in these systems. This gap may reflect the mechanistic complexity of enzymatic asymmetric catalysis and the difficulty of quantifying coupled solvent effects. Rather than acting through a single factor, DES may alter enzyme hydration, conformational ensembles, active-site interactions, substrate partitioning and mass-transfer behavior, thereby influencing the relative rates of competing stereochemical pathways and ultimately changing enantioselectivity. According to transition state theory, stereoselectivity fundamentally depends on the Gibbs free-energy difference between diastereomeric transition states [58], but fully resolving such transition states in DES-enzyme systems remains challenging.

Chen et al. proposed a machine-learning approach based on “quasi-transition states” (qTS) to predict enantioselectivity in asymmetric catalysis. By using only a small set of universal descriptors, this method enables efficient modeling of organocatalytic reactions while reducing computational cost [59]. Although currently applied to small-molecule organocatalysis, extracting descriptors from qTS rather than fully resolved transition states may provide a useful starting point for constructing QSPR models in DES-enzyme systems.

### 2.4 Extremozymes for DES-compatible biocatalysis

Many conventional enzymes show reduced activity or stability in media containing high proportions of DES, partly because their solvation environment and conformational dynamics are altered. As discussed above, the DES-to-water ratio changes the water activity, protein hydration state, and local distribution of DES components near the enzyme surface, thereby further affecting enzyme conformational motion [23,24,46].

These DES-related stresses partially overlap with the natural adaptations of some extremozymes. Halophilic proteins possess surface and solvation features that support function under highly ionic conditions [21,22], whereas thermophilic enzymes provide robust scaffolds for reactions conducted at elevated temperatures [60]. Marine extreme environments also constitute an important reservoir for extremozyme discovery. For example, enzymes recovered from deep-sea hypersaline environments have exhibited tolerance to high salinity, pressure, and polar solvents [61]. Such pre-existing robustness may broaden the range of DES compositions and aqueous fractions that can be tested without severe enzyme deactivation, thereby reducing the burden of solvent-side formulation optimization. For example, Haddadi et al. evaluated the thermophilic-like α-amylase from *Bacillus licheniformis* (BLA) and the related mesophilic α-amylase from *Bacillus amyloliquefaciens* (BAA) in several DES media. In 6 M Gly:ChCl (1:1), BLA showed higher activity and stability than BAA [62]. This comparison provides evidence that the pre-existing stability of a thermophilic enzyme may translate into improved tolerance under the same DES conditions.

However, systematic comparisons between extremozymes and conventional homologues under the same DES conditions remain scarce. Existing studies have primarily provided proof-of-feasibility that extremozymes can function in specific DES systems. For example, Álvarez et al. evaluated lipolytic enzymes from *Thermus thermophilus* HB27 in aqueous solutions of three ChDHP-based DES, among which ChDHP:EG (1:2) was the most compatible formulation. In a 2 M aqueous solution at 338.15 K, the enzymes retained more than 75% of their activity, and the standard Gibbs free energy of deactivation was 179.3 kJ mol^−1^, close to the value of 180.5 kJ mol^−1^ calculated in water [60].

Beyond their direct use as biocatalysts, extremozymes may serve as reference phenotypes for investigating DES tolerance. Recent enzyme-engineering studies show that secondary-structure transition regions and MD-derived residue-level distributions of water and DES components can guide mutation-site selection, while MD simulations can help explain the structural and solvation changes associated with improved variants [63,64]. These findings provide a rationale for comparing extremozymes with conventional homologues under matched DES conditions to identify structural and solvation features associated with DES tolerance.

Table 4 summarizes selected marine and extreme enzyme examples that are relevant to DES biocatalysis.

**Table 4:** Selected marine and extreme enzyme examples in DES biocatalysis.

Enzyme	DES composition	Aqueous phase	DES-induced effect	Reference
Halophilic cellulase	Gly:ChCl (1:2)	80% 50 mM citrate buffer (pH 4.8)	Saccharification of rice husk produced approximately 3-fold more glucose than the untreated sample	[65]
*Thermus thermophilus* lipase	ChDHP:EG (1:2)	2 mol L^−1^ ChDHP:EG in water	Significantly improved lipase performance under non-aqueous conditions	[60]
*Thermotoga maritima* AmyA	ChCl:urea (1:2)	60% DES; 30% water; 10% methanol	Achieved approximately 39 mM methyl glycosides with an increased alcoholysis/hydrolysis ratio	[25]
Marine *Streptomyces* lipase	ChCl:Gly (1:2)	No added water or buffer; substrates:NADES = 1:1	TAG content reached 55.8%, 1.38-fold higher than that in the solvent-free system	[66]
Marine *Janibacter* lipase	ChCl:sorbitol (1:1)	28.3% 20 mM phosphate buffer (pH 6.0)	Improved H_2_O_2_ tolerance in NADES, retaining 81.3% enzyme activity after 24 h	[67]

Note: EG: ethylene glycol; Gly: glycerol; ChDHP: cholinium dihydrogen phosphate; TAG: triacylglycerols.

## 3 Application of AI for DES-enzyme co-design in biocatalysis

To distinguish solvent-level prediction from enzyme-specific optimization, AI applications in DES-based biocatalysis can be organized into two connected levels according to their design objects and prediction targets. At the solvent level, AI-assisted DES design treats the formulation itself as the design object and is used to predict DES formation, formulation-level physicochemical properties, or candidate compositions from information on the components, their ratios, and relevant conditions. At the system level, AI-driven DES-enzyme co-design focuses on the response of a specific enzyme to a defined DES environment. It therefore extends the prediction target from solvent properties to enzyme activity, stability, selectivity, or process performance and requires the relationship between DES formulations, enzymes, and reaction conditions to be considered.

Accordingly, Section 3.1 discusses AI-assisted DES design, including DES formation screening, physicochemical-property prediction, and candidate-formulation design. Section 3.2 then examines AI-driven strategies for DES-enzyme co-design. It first evaluates existing enzyme-specific DES screening and performance-prediction studies and then discusses how enzyme information, including sequence, structure, flexibility, stability, and the catalytic microenvironment, may be incorporated into computational frameworks for DES-enzyme matching and joint optimization. The DES properties, enzyme-level factors, and their integration across these two design levels are summarized in Fig. 3.

**Fig. 3:**
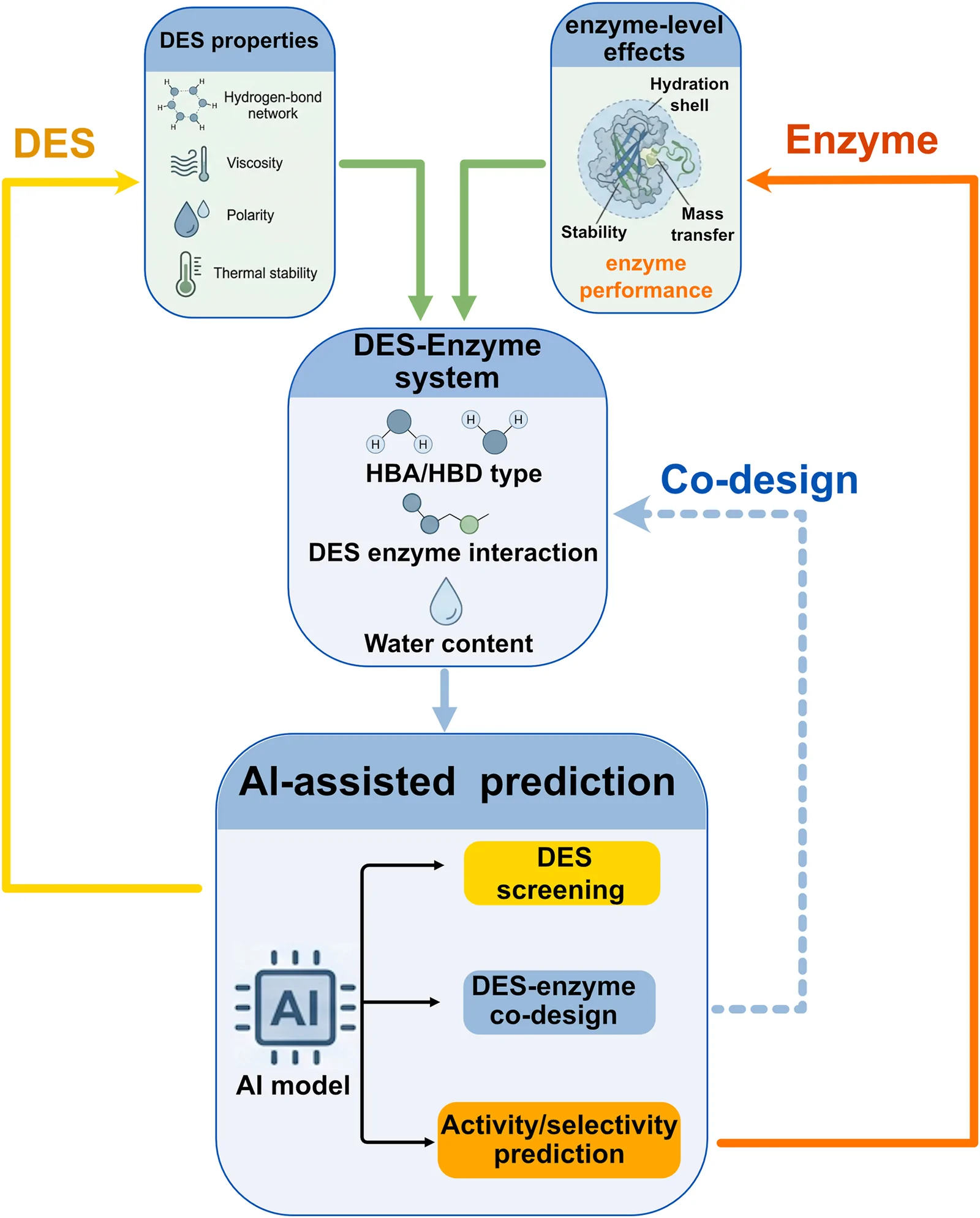
AI-assisted DES-enzyme co-design for rational biocatalytic system design.

### 3.1 AI-assisted DES design

At the solvent level, current AI applications address three complementary tasks: determining whether candidate components can form a DES, predicting the physicochemical properties of a specified formulation, and proposing candidate formulations subject to target properties. As discussed in Section 2.1, reported inputs for DES-formation prediction include MD-derived descriptors of hydrogen-bond numbers and lifetimes and canonical SMILES representations encoding component structures and stoichiometric ratios. The applied machine-learning algorithms include conventional classifiers such as extra trees and an ELECTRA-based Transformer binary classifier, which were used to determine whether candidate components and ratios could form stable DES [38,39].

Once a candidate formulation has been defined, QSPR models can connect its composition and relevant conditions with formulation-level properties. COSMO-RS-derived σ-profiles and related descriptors are frequently used for this purpose. This model uses quantum-chemical calculations to generate σ-profiles that represent the distribution of screening charge density over the surfaces of DES components [68]. By encoding information related to specific intermolecular interactions, COSMO-RS-derived descriptors can improve machine learning (ML)-based property prediction even when experimental datasets are limited [69]. For example, Lemaoui et al. developed a high-fidelity QSPR model using COSMO-RS descriptors and multiple linear regression (MLR) to predict the density and viscosity of hydrophobic eutectic solvents (HES), achieving exceptional accuracy (R^2^ ≥ 0.99) [70]. Similarly, artificial neural networks (ANN) are also frequently combined with COSMO-RS descriptors to construct QSPR models for predicting DES viscosity [71,72]. Cysewski et al. combined these descriptors with a neural network algorithm to construct a QSPR model for predicting theophylline solubility in various NADES, thereby accelerating high-throughput solvent screening [73].

AI-assisted DES design can also proceed from forward prediction to candidate generation. Luu et al. developed diffusion and Transformer-based models for forward and inverse molecular-design tasks. After pretraining on the QM9 dataset and further training on DES-related tasks, their multi-task generative model proposed several candidate combinations with targeted DES-related properties [74]. Together, these approaches can reduce the initial formulation space by identifying feasible combinations, estimating their properties, and proposing new candidates. Their outputs nevertheless remain solvent-level endpoints; whether a selected formulation creates a suitable environment for a particular enzyme must be evaluated at the DES-enzyme system level.

### 3.2 AI-driven strategies for DES-enzyme co-design

At the system level, the prediction target changes from a DES property to an enzyme-specific response. Existing studies have begun to establish this mapping. Logarušić et al. used PLR models to relate DES physicochemical properties and COSMO-RS mixture descriptors to FDH activity and stability and to NADH stability [18]. Radović et al. calculated COSMO-RS mixture descriptors incorporating the HBA, HBD, and water and developed separate ANN models for predicting the inactivation rate constants of three dehydrogenases [17]. Sosa et al. used five COSMO-RS descriptors for each of the HBA, HBD, and water to predict the relative activity of laccase in aqueous DES media [30]. These studies use enzyme performance rather than a solvent property as the model output. However, they were developed for a fixed enzyme or through separate enzyme-specific models, meaning that enzyme identity was represented implicitly by the corresponding dataset rather than explicitly through protein descriptors.

The way in which an enzyme should enter the computational framework therefore depends on the intended scope of the model. When formulations are screened for one fixed enzyme, the enzyme does not vary within the dataset, and a model can directly learn the relationship between DES and process descriptors and the measured response. Such a model can rank formulations for that enzyme, but its applicability to another enzyme cannot be assumed. In contrast, cross-enzyme matching or co-design requires the enzyme to become an explicit input so that differences among enzyme families, homologues, or engineered variants can be distinguished.

Enzyme information may be represented at several levels. Sequence-level features can distinguish enzyme identity, evolutionary relationships, and mutation patterns that may be associated with DES tolerance. Structural features can describe the interface encountered by the solvent more directly, including surface charge and hydrophobicity, solvent-accessible regions, the spatial distribution of surface residues, and active-site geometry.

These enzyme features need to be paired with formulation and operating variables. The solvent-side representation may include HBA and HBD identity and molar ratio, aqueous fraction or water activity, pH, polarity, viscosity, and interaction descriptors. Temperature, incubation and reaction time, substrate loading, cofactor conditions, and immobilization state further define the experimental context of activity, stability, selectivity, conversion, or productivity measurements. Together, these enzyme, solvent, and operating variables define the catalytic microenvironment experienced by the enzyme.

Within this framework, current AI studies primarily perform enzyme-specific DES screening or DES-enzyme matching. Genuine co-design would additionally treat both the DES formulation and the enzyme or its variants as variable design objects and evaluate their combinations against defined catalytic and process endpoints. Although a complete implementation has not yet been demonstrated for DES-enzyme systems, these tasks could ultimately be connected through a design-build-test-learn cycle in which experimental measurements update the formulation- and enzyme-level models used to select the next design round [75,76].

## 4 Current bottlenecks and future perspectives

Despite their demonstrated potential in biocatalysis, the rational design of DES–enzyme systems faces two interrelated bottlenecks. First, the vast combinatorial space of DES formulations, enzyme variants, water contents, and operating conditions, together with their coupled effects on enzyme performance, makes optimization on either the solvent or enzyme side alone insufficient [18,31]. Second, available DES–enzyme datasets are limited and heterogeneous and sparsely cover this joint design space, restricting model transferability and making the reliable training and external validation of end-to-end co-design models difficult. Addressing these bottlenecks will require coordinated advances in DES-side formulation design and enzyme-side engineering, followed by their integration at the system level.

On the solvent side, current ML-based QSPR models mainly perform forward prediction of individual DES properties. Future DES engineering should move toward inverse design constrained by multiple target properties. As discussed in section 3.1, Luu et al. provided an early example by combining property prediction with generative design to propose DES candidates with targeted properties [74].

Alongside DES formulation design, the enzyme itself can be adapted to the target medium. As discussed in section 2.4, recent enzyme-engineering studies have shown that DES tolerance can be improved by modifying protein solvation and conformational features [63,64]. These studies establish the feasibility of enzyme-side adaptation but optimize the enzyme against a selected DES environment. Future DES-enzyme co-design should instead treat the DES formulation and the enzyme sequence as coupled design variables and evaluate their combinations using enzyme-specific catalytic and process endpoints.

Extremozymes can contribute to this strategy by providing reference phenotypes from which potentially transferable adaptation features can be learned. The recently reported HaloMPNN model illustrates this principle on the enzyme side. By retraining ProteinMPNN on proteins from “salt-in” extreme halophiles, HaloMPNN redesigned non-halophilic proteins toward lower predicted isoelectric points, greater surface acidity, and reduced surface and core hydrophobicity, thereby shifting their predicted properties toward those of natural halophilic proteins [77]. This study shows that retraining a general protein-design model using datasets of proteins with clearly defined environmental adaptations can transfer the corresponding sequence and surface features to non-extremophilic protein scaffolds. For DES-enzyme systems, MPNN models retrained using data from different types of extremozymes could provide enzyme-side design priors related to adaptation to ionic environments, hydration maintenance, or conformational robustness, thereby providing a rational starting point for subsequent DES-enzyme co-design that incorporates specific DES formulations and experimental performance endpoints.

Models based on Transformer or message-passing neural network (MPNN) architectures can support representation learning and candidate generation for DES formulations and enzyme sequences. For the prediction of process performance, QSPR models could further combine MD- or COSMO-RS-derived descriptors with predictive algorithms to incorporate information related to solvent dynamics and component interactions. However, current enzyme-performance models are generally developed for a fixed enzyme, leaving enzyme identity implicit and thereby limiting their transferability to new enzyme–formulation combinations. Moreover, as noted above, the limited scale and heterogeneity of available DES–enzyme datasets further constrain the reliable training and external validation of end-to-end models. Therefore, a more feasible near-term strategy is to couple models with complementary functions: a DES-side model proposes candidate formulations, an enzyme-side model proposes sequence variants, and a system-level predictor or multiscale simulation evaluates the resulting formulation–enzyme pairs. Experimental measurements can then continually update each model through active learning within a design–build–test–learn cycle, thereby connecting DES formulation optimization and enzyme engineering in a co-design workflow tailored to a specific reaction system.

## 5 Conclusion

DES have considerable potential to advance non-aqueous biocatalysis by providing tunable microenvironments that modulate enzyme activity, stability and selectivity. This review has summarized recent applications of DES in biocatalysis and examined the emerging role of AI in rational design of DES-enzyme systems. Current computational approaches are gradually moving beyond the prediction of individual DES properties toward the screening of formulations based on enzyme-level responses.

Future progress will require integrated strategies that jointly consider DES formulation features and enzyme physicochemical and structural requirements. By combining solvent engineering, protein engineering and AI-driven modeling, DES-based biocatalysis may move toward more rational and sustainable catalytic systems.

## Declaration of competing interest

The author is an Editorial Board Member/Editor-in-Chief/Associate Editor/Guest Editor for this journal and was not involved in the editorial review or the decision to publish this article.

The authors declare the following financial interests/personal relationships which may be considered as potential competing interests: Given his role as Associate Editor, Jifeng Yuan had no involvement in the peer review of this article and had no access to information regarding its peer review. Full responsibility for the editorial process for this article was delegated to another journal editor.

## Abbreviations

**AI:** Artificial intelligence; **ANN:** Artificial neural networks; **ChCl:** Choline chloride; **ChDHP:** Cholinium dihydrogen phosphate; **COSMO-RS:** Conductor-like screening model for real solvents; **DES:** Deep eutectic solvents; **DMSO:** Dimethyl sulfoxide; **DMY:** Dihydromyricetin; **EG:** Ethylene glycol; **FDH:** Formate dehydrogenase; **HBA:** Hydrogen bond acceptor; **HBD:** Hydrogen bond donor; **HRP:** Horseradish peroxidase; **Gly:** Glycerol; **HES:** Hydrophobic eutectic solvents; **IL:** Ionic liquids; **MD:** Molecular dynamics; **ML:** Machine learning; **MLP:** Multilayer perceptron; **MLR:** Multiple linear regression; **MPNN:** Message passing neural network; **NADES:** Natural deep eutectic solvents; **PA:** Phosphatidic acid; **PC:** Phosphatidylcholine; **PLD:** Phospholipase D; **PLR:** Piecewise linear regression; **QSPR:** Quantitative structure-property relationships; **RDES:** Reactive deep eutectic solvent; **VOC:** Volatile organic compounds.
